# Trends in Homelessness and Social Sustainability: Veterans vs. Non-Veterans in the US

**DOI:** 10.3390/healthcare13090967

**Published:** 2025-04-22

**Authors:** Ângela Leite, Catarina Vieira da Silva

**Affiliations:** Center for Philosophical and Humanistic Studies, Faculty of Philosophy and Social Sciences, Universidade Católica Portuguesa, 4710-302 Braga, Portugal; crsilva@ucp.pt

**Keywords:** homeless persons, homeless veterans, homelessness, mental disorders, substance-related disorders, substance use disorders, sustainability, veterans

## Abstract

**Introduction**: Social sustainability is deeply connected to the well-being of marginalized groups, and it is important to highlight how mental health impacts the social inclusion of homeless individuals, particularly veterans. Homelessness is a growing global issue, disproportionately affecting U.S. veterans, with mental health challenges playing a significant role in its onset and perpetuation. Purpose: This study aims to compare the sociodemographic and clinical characteristics of homeless veterans and non-veterans in the U.S. **Method**: Using public data (*N* = 6295), this quantitative study applies descriptive and inferential statistical analyses. **Results**: Homeless veterans are more likely than non-veterans to be older, male, and identify as Caucasian or African American. They are more frequently high school graduates or have higher education, and report being divorced, widowed, married, or in varied employment statuses (full-time, part-time, or unemployed). Veterans exhibit higher rates of severe mental illnesses, schizophrenia, trauma- and stressor-related disorders, ADHD, bipolar disorder, personality disorders, depression, anxiety, and substance or alcohol use disorders. However, they are less likely than non-veterans to report substance-induced disorders, intoxication, dependence, or abuse involving cocaine, cannabis, opioids, and other substances. **Conclusions**: Psychosocial interventions for homeless veterans should prioritize mental health-related concerns, whereas efforts for homeless non-veterans should focus on addressing substance use. Future research should develop tailored interventions, explore the sociodemographic factors influencing homelessness, and investigate the interplay between trauma, mental health, and substance use. Addressing these issues can contribute to a more resilient, inclusive, and sustainable society by providing long-term support and integration opportunities for those most affected. The novelty of this study lies in distinguishing between mental health issues prevalent in veterans and substance use disorders more common in non-veterans, offering insights for tailored interventions. It also connects these findings to social sustainability, suggesting that addressing these issues can promote a more inclusive and resilient society.

## 1. Introduction

Homelessness is a growing global concern that affects both physical and mental health [1]. Research indicates that homelessness is correlated with heightened levels of mental illness [2], with mental health issues often serving as a catalyst for ongoing homelessness [3]. The literature highlights the significant overlap between homelessness, mental health issues, and substance abuse among homeless individuals, particularly veterans. While much research has focused on homelessness in general, few studies have specifically examined the differences between homeless veterans and non-veterans. Understanding the distinct characteristics of these subgroups is crucial for designing effective interventions and providing targeted support.

Homelessness is often defined by a lack of adequate housing, ranging from living in shelters or on the streets to temporary or insecure accommodations. The European Observatory on Homelessness and the European Federation of National Organizations [4] proposed the ETHOS typology, classifying homelessness into four categories: roofless, houseless, insecure, and inadequate accommodation. Similarly, the U.S. Department of Veterans Affairs [5] defines homelessness as a lack of stable housing, including living in emergency shelters or transitional housing. Homeless veterans face challenges linked to military service, including psychological trauma, physical injuries, and reintegration difficulties [6]. Mental health issues like PTSD, depression, and substance abuse are prevalent and contribute to their vulnerability [7]. The prevalence of homelessness among veterans is alarming, with many experiencing extended periods of homelessness [8]. Studies show homeless veterans are more likely to have severe mental health conditions and substance abuse compared to non-veterans [9]. Despite growing recognition of these challenges, research comparing homeless veterans with homeless non-veterans is limited. Most studies focus on broad comparisons, overlooking the unique experiences of homeless veterans. This study aims to address this gap by examining sociodemographic and clinical differences between homeless veterans and non-veterans in the U.S. It seeks to identify factors contributing to severe mental illness and emotional disorders among homeless veterans and provide insights for developing more effective, tailored interventions.

### 1.1. Homelessness Concept

The European Observatory on Homelessness and the European Federation of National Organizations [4] introduced the ETHOS definition and typology of homelessness and housing exclusion. ETHOS includes thirteen categories covering twenty-four living situations, grouped into four main headings: roofless, houseless, insecure, and inadequate accommodation. Roofless and houseless define homelessness, while insecure and inadequate pertain to housing exclusion. Homelessness is defined as living in inadequate housing or lacking access to adequate housing [10]. This study uses data from the U.S. Substance Abuse and Mental Health Services Administration (SAMHSA) and the Center for Behavioral Health Statistics and Quality [11], which define homelessness as having no fixed address, including those in shelters. The U.S. Department of Veterans Affairs (VA) [5] defines homelessness as lacking a fixed, regular, and adequate nighttime residence, including those in shelters, transitional housing, or places not intended for habitation. Homelessness reflects social exclusion and vulnerability to systemic barriers like discrimination or lack of resources [12]. This definition covers visible and hidden homelessness, including precarious or temporary accommodations. Henry et al. [13] also associate homelessness with individuals having a diagnosable disability and being continuously homeless for a year or experiencing four or more episodes within three years.

Homelessness in the U.S. is defined by HUD [5] as lacking a permanent living arrangement, with individuals or families considered transient. Kuhn and Culhane [14] identified three groups of homeless shelter users: (1) transitionally homeless, with brief and infrequent shelter stays and minimal mental health or substance abuse issues; (2) episodically homeless, with frequent, short-term shelter stays and higher rates of mental illness and substance abuse, often cycling through shelters, hospitals, and prisons; and (3) chronically homeless, with long-term homelessness and high prevalence of mental illness and substance abuse, using shelters as a substitute for institutional care [15]. Culhane [16] emphasizes that long-term homelessness involves persistent barriers like mental illness, substance abuse, and physical disabilities. He advocates for permanent housing models, such as “Housing First”, which prioritize stable housing before addressing other issues [17]. Culhane and colleagues [18] stress the importance of sustainable solutions, including expanding affordable housing and improving access to mental health and addiction services, to prevent and address chronic homelessness [18].

Smith and Castañeda [9] challenged the stereotype that the majority of homeless individuals suffer from severe mental illness, which they argue limits access to services. They identified two key beliefs that contribute to this stereotype: (1) A large proportion of people experiencing homelessness originate from the deinstitutionalization of individuals from mental health facilities, and (2) mental illness is more prevalent among people at risk of homelessness compared to the housed population. Despite an increasing number of homeless women, the majority of people experiencing homelessness continue to be male [19].

### 1.2. Homeless Veterans

Homeless veterans are individuals who have served in the armed forces and are currently without adequate housing [20]. Most homeless veterans are men [21,22] and suffer from the psychological effects of war [23]. In the U.S., veterans make up a significant portion of the homeless population. Wood et al. [24] found that veterans constitute 5.6% of rough sleepers in Australia, spending an average of 6.3 years homeless. Homeless veterans are four times more likely to use emergency health departments than non-veteran homeless individuals [21,25,26]. Factors contributing to homelessness include inadequate affordable housing [25], limited mental health services, high unemployment, economic crises, and individual issues like childhood trauma, poverty, low education, health conditions, combat stress, psychiatric disorders, and substance abuse [6].

### 1.3. Homeless Veterans and Mental Health

Mental illnesses affect a person’s thinking, mood, and behavior, ranging from mild to severe [27]. Serious mental illness (SMI) interferes with life and functioning, including schizophrenia, bipolar disorder, major depression, and severe anxiety disorders. SMI is defined by three criteria: a psychiatric diagnosis (DSM-5 or ICD) [28], illness duration over two years with functional disability [29]), and disability in functioning [30]. A person with a serious emotional disturbance (SED) exhibits one or more significant behavioral or emotional challenges over an extended period that adversely affect their functioning. These may include difficulties with learning not linked to other conditions, trouble maintaining relationships, inappropriate behaviors or feelings, persistent depression, or physical symptoms tied to personal issues [11,31].

Veterans may face mental health issues and substance abuse due to a lack of social support, loneliness, and barriers to treatment, contributing to chronic homelessness [32]. Mental health issues are more common among veterans (35%) than non-veterans (29.1%) [24]. Homeless veterans experience higher rates of physical, mental, and social challenges, including difficulties transitioning to civilian life. Nichter et al. [8] found a 10.2% lifetime prevalence of homelessness among U.S. veterans, often starting a decade after discharge, linked to adverse childhood experiences, trauma, and mental health issues. Homeless veterans are at higher risk of suicide and mental health disorders, emphasizing the need to address early-life adversities. Ding et al. [33] found that 76.7% of homeless veterans had a mental disorder, 47.4% had drug-related conditions, and 37.2% had co-occurring disorders (COD). Veterans with COD had lower mental health and empowerment scores. Smartt et al. [34] noted that homelessness can worsen mental illness, especially with substance misuse. Factors like transitioning to civilian life, relationship and employment challenges, mental health issues, and adverse events contribute to veterans’ homelessness [35]. Crone et al. [36] highlighted disproportionate homelessness among African American veterans due to systemic distrust and limited service access, recommending tailored outreach and inclusion in policymaking. Tsai and Kelton [37] observed that only a quarter of homeless veterans used available services due to stigma, lack of awareness, and transportation issues, urging improved awareness and care coordination.

### 1.4. This Study

People at risk of homelessness are a very heterogeneous population, and therefore, it is important to understand the specificities of the subgroups that make up this population in order to intervene successfully. Most studies on homelessness focus on comparisons with non-homeless people or differences in gender, age, and other sociodemographic variables. The same is true for studies on veterans that compare them with non-veterans. However, few studies compare homeless veterans with homeless non-veterans. To fill this gap, this study aims to identify the sociodemographic and clinical differences between American homeless veterans and homeless non-veterans and to determine factors associated with severe mental illness and emotional disorders. It is expected that this study may provide data-driven insights that support differentiated approaches to preventing and addressing homelessness, mental illness, and substance use among distinct subgroups.

## 2. Methods

### 2.1. Procedures

The data used in this study were provided by the Center for Behavioral Health Statistics and Quality, Substance Abuse and Mental Health Services Administration (SAMHSA), USA [11], under the designation “Mental Health Client-Level Data (MH-CLD): 2019”. MH-CLD is a public database that provides demographic and mental health characteristics for clients who have used mental health services in facilities that report to individual state administrative data systems.

Data were collected from the following states: Alabama, Alaska, Arkansas, California, Colorado, Connecticut, Delaware, District of Columbia, Florida, Hawaii, Idaho, Illinois, Indiana, Kentucky, Louisiana, Maryland, Massachusetts, Michigan, Minnesota, Mississippi, Missouri, Montana, Nebraska, Nevada, New Jersey, New Mexico, New York, North Carolina, North Dakota, Ohio, Oklahoma, Oregon, Pennsylvania, Rhode Island, South Carolina, South Dakota, Tennessee, Texas, Utah, Vermont, Virginia, Washington, Wisconsin, Wyoming, and Puerto Rico.

The entire database included 6,362,044 participants; however, only homeless participants were selected (based on the residential status variable, with response options: 1-homeless, 2-private residence, 3-other), along with veterans and non-veterans, resulting in 102,656 participants. It is worth noting that this study only includes homeless individuals (both veterans and non-veterans) who have sought mental health services.

### 2.2. Material

The variables chosen from the MH-CLD were as follows.

#### 2.2.1. Sociodemographic Variables

Sociodemographic variables included gender (1: male, 2: female), age (14 groups with 5-year intervals), marital status (1: never married, 2: married, 3: separated, 4: divorced, 5: widowed), race (1: American Indian/Alaska Native, 2: Asian, 3: Black or African American, 4: Native Hawaiian or other Pacific Islander, 5: White, 6: some other race alone/two or more races), education (1: special education, 2: 8 years or less, 3: 9–11 years, 4: 12 years or GED (General Educational Development Test), 5: 13 years or more), competitive employment status (a type of employment where an individual is hired based on their qualifications, skills, and ability to perform the job; 1: full-time, 2: part-time, 3: employed full-time/part-time not differentiated, 4: unemployed, 5: not in labor force), and veteran status (1: yes, 2: no), where 1 (yes) means a person who served in the active military, naval, or air service and was discharged or released under conditions other than dishonorable.

#### 2.2.2. Diagnosis and Clinical Variables

Diagnosis and clinical variables included SMI/SED status (1: SMI, 2: SED and/or at risk for SED, 3: not SMI/SED); mental health diagnoses one, two, three, and other (1: trauma- and stressor-related disorders, 2: anxiety disorders, 3: attention deficit/hyperactivity disorder [ADHD], 4: conduct disorders, 5: delirium, dementia, 6: bipolar disorders, 7: depressive disorders, 8: oppositional defiant disorders, 9: pervasive developmental disorders, 10: personality disorders, 11: schizophrenia or other psychotic disorders, 12: alcohol or substance use disorders, 13: other disorders/conditions, namely, eating disorders (e.g., anorexia, bulimia), sleep disorders (e.g., insomnia, narcolepsy), or neurocognitive disorders); number of mental health diagnoses reported; substance use diagnosis (1: alcohol-induced disorder, 2: alcohol intoxication, 3: substance-induced disorder, 4: alcohol dependence, 5: cocaine dependence, 6: cannabis dependence, 7: opioid dependence, 8: other substance dependence, 9: alcohol abuse, 10: cocaine abuse, 11: cannabis abuse, 12: opioid abuse, 13: other substance-related conditions); and substance use problem (1: yes, 2: no).

### 2.3. Data Analysis

Descriptive analysis indicators were used to characterize the sample. Differences were assessed using a *t*-test for continuous variables and a chi-squared test for nominal variables. Spearman correlations were used to evaluate the relationship between variables, and only the significant ones entered the logistic regression. Binary logistic regression was employed to identify variables influencing serious mental illness and veteran status.

All assumptions were tested, including the following: assumption of normality (skewness ± 2 and kurtosis ± 7), assumption of homogeneity of variance (Levene’s test not significant), assumption of homogeneity of variance-covariance matrices (Box’s M test not significant), assumption of appropriate outcome structure (binary logistic regression requires the dependent variable to be binary), assumption of observation independence (observations should not come from repeated measurements or matched data), assumption of the absence of multicollinearity (Durbin–Watson test between 1.5 and 2.5), assumption of linearity of independent variables (Cook’s D < 4) and log odds, and assumption of a large sample size. The significance threshold is set at 0.05 (5%). All analyses were carried out using IBM SPSS, 28th edition.

## 3. Results

### 3.1. Descriptives and Differences

The sample of this study consists of 102,656 homeless Americans aged 18 or older. Most are white males aged between 25 and 54 years. More than half of the sample have never been married, and more than half have more than nine years of education. A large percentage are unemployed or not in the labor force, including those who are retired, disabled, students, or in other categories. Among the sample, 6% have veteran status (Table 1).

Veterans (N = 6295) are significantly older (M = 9.70; SD = 2.60) than non-veterans (N = 96,361) (M = 8.80; SD = 2.53) [t(7096, 501) = 26.73; *p* < 0.001; d = 0.37] (here, the mean concerns the different age levels). There are statistically significant differences [χ^2^(1) = 879.29; *p* < 0.001; Φ = 0.09] in the gender distribution based on war veteran status, with significantly more men in the veterans’ group (78.2% men vs. 21.8% women) compared to the non-veterans’ group (59.3% men vs. 40.7% women).

There are statistically significant differences [χ^2^(6) = 162.42; *p* < 0.001; Φ = 0.04] in the distribution of veteran status by race, with fewer American Indian or Alaska Native and fewer Asian individuals in the veterans’ group compared to the non-veterans group. However, there are more White individuals in the veterans’ group than in the non-veterans’ group. There are no significant differences for the other racial categories.

There are statistically significant differences [χ^2^(5) = 833.60; *p* < 0.001; Φ = 0.09] in the distribution of veteran status by education, with veterans having more years of education than non-veterans. Most homeless individuals are single. The difference between homeless veterans and non-veterans in marital status is significant only for the “divorced, widowed” category, with veterans being divorced/widowed (i.e., without a partner) more than non-veterans [χ^2^(4) = 451.96; *p* < 0.001; Φ = 0.07].

Concerning competitive employment status, significant differences were found in relation to unemployment and being out of the workforce. Homeless veterans are significantly more likely to be unemployed than homeless non-veterans. Moreover, non-veterans are significantly more likely to be out of the workforce than homeless veterans [χ^2^(5) = 445.64; *p* < 0.001; Φ = 0.07].

Regarding diagnoses 1, 2, and 3 (Table 2), the number of missing cases increases exponentially from diagnosis 1 to 2, and from 2 to 3, with over 90% of cases missing in diagnosis 3. This indicates that the likelihood of the same person receiving a second diagnosis is small, and even smaller for a third diagnosis. Most of the sample has a serious mental illness. Among these, depressive disorders are the most prevalent in diagnosis 1, while anxiety disorders are most common in diagnoses 2 and 3.

The least prevalent conditions in diagnosis 1 are pervasive developmental disorders, oppositional defiant disorders, and conduct disorders. In diagnosis 2, conduct disorders and delirium are the least common, and in diagnosis 3, delirium, oppositional defiant disorders, pervasive developmental disorders, and conduct disorders remain the least reported. However, most individuals in the sample have only one diagnosis.

Concerning substance use diagnoses, most of the sample exhibits substance use problems, with opioid dependence and alcohol dependence being the most prevalent, while alcohol-induced disorders are the least common (Table 2).

When comparing homeless veterans and non-veterans, veterans have more severe mental illnesses but less serious emotional disturbances than non-veterans. In diagnosis 1, veterans are more likely to report depressive disorders and schizophrenia or other psychotic disorders, while non-veterans have higher frequencies of alcohol or substance use disorders (Table 2). About 66.2% of the sample does not have a second diagnosis. Among those who do, non-veterans show higher frequencies across all disorders compared to veterans, except for schizophrenia or other psychotic disorders (Table 2). In diagnosis 3, non-veterans exhibit higher frequencies in all disorders compared to veterans, although 91% of cases are missing.

With respect to the number of mental health diagnoses reported, veterans are more likely to have zero or one diagnosis, whereas non-veterans are more likely to have two or three diagnoses (Table 2). Veterans report more substance use problems than non-veterans. However, non-veterans report higher frequencies of alcohol intoxication, substance-induced disorders, alcohol dependence, cocaine dependence, cannabis dependence, opioid dependence, other substance dependencies, alcohol abuse, cocaine abuse, cannabis abuse, opioid abuse, and other substance-related conditions (Table 2).

In Table 3, the disorders reported by homeless individuals are presented. Depressive disorder was the most frequently reported, followed by trauma- or stressor-related disorders, schizophrenia or other psychotic disorders, alcohol- or substance-related disorders, and bipolar disorder. The least reported disorder was oppositional defiant disorder (Table 3). Veterans reported more depressive disorders and schizophrenia or other psychotic disorders than non-veterans. All other disorders were more frequent in non-veterans than in veterans (Table 3).

### 3.2. Demographic Characteristics

American homeless veterans are predominantly male, aged between 30 and 59, white, with 12 years of education or more, never married, and unemployed or not in the labor force. Most of this sample presents with severe mental illness, with the vast majority having only one diagnosis. Regarding diagnosis one, the subsample shows primarily depressive disorders, followed by schizophrenia or other psychotic disorders. For diagnosis two, alcohol- or substance-use disorders, followed by trauma- and stressor-related disorders and anxiety disorders, were the most frequent pathologies. Concerning diagnosis three, alcohol- or substance-use disorders were the most prevalent, with most of the sample presenting a substance use problem, and 4.8% reporting alcohol dependence (Table 2 and Table 3).

### 3.3. Logistic Regression

A binary logistic regression with sociodemographic and clinical variables as predictors and war veteran status as the outcome variable was conducted on the full sample. Table 4 shows the results obtained from this logistic regression model, with all variables defined as categorical (including the dependent variable), except for age and number of diagnoses. The general model fit was statistically significant (χ^2^(30) = 5521.17; *p* < 0.001) compared to the null model. Finally, the model showed a low level of explained variance (Nagelkerke R^2^ = 0.14), with a 93.9% correct classification rate.

The results (Table 4) highlight the variables that significantly contribute to explaining war veteran status. Veterans are more likely to be male, older than non-veterans, White, Black or African American, or identify as having two races. Additionally, veterans are more likely to have 12 or more years of education. They are also more likely to be divorced, widowed, or married. Veterans are more likely than non-veterans to be employed full-time or part-time or to be unemployed (Table 4).

Veterans are also more likely to have a serious mental illness, a serious emotional disturbance, and/or to be at risk for serious emotional disturbance (SED) than non-veterans. Regarding mental health diagnoses, veterans are more likely to have trauma- and stressor-related disorders and schizophrenia or other psychotic disorders in diagnosis 1. However, in diagnosis 2, veterans are less likely than non-veterans to present with trauma- and stressor-related disorders, anxiety disorders, attention-deficit/hyperactivity disorder, bipolar disorders, depressive disorders, personality disorders, schizophrenia or other psychotic disorders, and alcohol or substance use disorders. Similarly, in diagnosis 3, veterans are less likely than non-veterans to present with personality disorders (Table 4).

Furthermore, veterans are less likely to report alcohol-induced disorders, alcohol intoxication, substance-induced disorders, alcohol dependence, cocaine dependence, cannabis dependence, opioid dependence, other substance dependencies, alcohol abuse, cocaine abuse, cannabis abuse, opioid abuse, and other substance-related conditions than non-veterans (Table 4).

## 4. Discussion

This research aimed to examine the sociodemographic and clinical differences between homeless veterans and homeless non-veterans, as well as to identify factors associated with severe mental illness and severe emotional disorders. Publicly available data were utilized, revealing distinct profiles for veterans and non-veterans. These findings have significant implications for evidence-based social service delivery.

Research on homeless veterans and non-veterans highlights the critical importance of developing equitable, inclusive, and supportive systems to address both immediate needs and long-term well-being [7]. The distinct challenges faced by veterans—particularly higher rates of PTSD, mental health conditions, and substance use—necessitate specialized, targeted policies and interventions [38,39]. Tailored services that consider the unique sociocultural and clinical characteristics of both groups are essential for providing access to critical resources, fostering recovery, and ensuring stability [40,41].

The aging veteran population, predominantly male, faces an increased risk of homelessness, especially among veterans aged 35–44 [21,26]. Tsai et al. [42] report over 6000 homeless male veterans compared to 700 female veterans, highlighting the need for targeted interventions that address the specific challenges of aging male veterans. These interventions should focus on promoting long-term housing stability, access to essential services, and chronic condition management. As the veteran population continues to age, policies must prioritize long-term care, particularly for chronic conditions like PTSD and depression, with a focus on integrated mental health and substance use prevention [7,8]. Preventative strategies that combine chronic pain management with mental health support can help mitigate substance use and improve overall well-being [43,44].

Social sustainability requires proactive planning for demographic shifts, particularly in supporting elderly, disabled, and chronically ill veterans. Adequate healthcare and social services must be in place to meet their needs without overwhelming existing systems [45]. Policies should emphasize sustained, accessible care and community-based support [3] to prevent further marginalization. Additionally, racial disparities in homelessness and healthcare access necessitate race-conscious policy solutions, with Otiniano Veríssimo et al. [46] identifying being white as a risk factor for homelessness and Olivet et al. [47] noting heightened risks for Black veterans. Targeted outreach and culturally competent support services are crucial for promoting equitable housing programs and addressing the distinct needs of these vulnerable veteran populations.

Employment also plays a critical role in social sustainability. Veterans tend to have more years of education than non-veterans [26,48], which presents an opportunity to invest in workforce development programs that leverage veterans’ skills and training. However, veterans still face barriers to employment, including skills mismatches and employer discrimination [12,49]. Veterans are more likely to be employed or unemployed rather than out of the workforce, whereas non-veterans are more often not working due to retirement or disability [50,51]. This trend underscores the importance of job placement programs, career transition support, and policies that reduce employment instability. Inclusive employment initiatives, workforce training, and employer education can promote economic security, reduce homelessness risk, and foster long-term stability [38,39].

Incorporating mental health support into housing programs is also essential for enhancing long-term recovery and stability [40,41]. Service providers should receive cultural competency training to better address the unique experiences of veterans [52], while inclusive housing and employment strategies ensure veterans’ economic inclusion and community integration.

Veterans’ higher likelihood of being divorced, widowed, or previously married [48] underscores the role of social support structures in promoting well-being. The higher rates of divorce and widowhood may also signal increased vulnerability to social isolation, making relationship support programs and community engagement initiatives critical for fostering long-term resilience. Strengthening social connections through targeted interventions can contribute to the overall stability and well-being of veterans, thus supporting social sustainability [32].

Mental health remains a central issue, particularly for veterans experiencing PTSD and schizophrenia, which require sustained mental health support [8]. While veterans experience fewer comorbid mental health conditions than non-veterans, the high prevalence of PTSD and depression calls for specialized, trauma-informed care and early intervention strategies [53]. It is worth noting that many individuals in the non-veteran cohort have also experienced various forms of trauma and complex challenges, including incarceration [54]. Incarceration can worsen mental health by exposing individuals to chronic stress, isolation, and often violent or dehumanizing conditions [55]. Limited access to quality mental health care within prisons, along with disrupted treatment and social support, can lead to the deterioration of existing conditions or the onset of new disorders [54]. Upon release, barriers to housing, employment, and continued care further compound psychological distress [55]. Expanding access to mental health services tailored to veterans’ unique experiences—such as peer support networks, veteran-specific rehabilitation programs, and trauma-informed therapy—can foster long-term well-being and successful community reintegration [6].

Moreover, veterans’ lower rates of substance- and alcohol-induced disorders suggest that existing support structures, such as VA programs and military discipline, may play a protective role [56]. Strengthening these programs and integrating substance use treatment with mental health care can further improve health outcomes. Early intervention and harm reduction strategies are essential in addressing substance use concerns, as these challenges persist across both veteran and non-veteran populations [33].

Preventative measures—ranging from early mental health interventions to substance use prevention programs—are integral to reducing long-term social costs and building a resilient society [7]. Social sustainability depends on comprehensive, integrated healthcare systems that promote long-term stability for both veterans and non-veterans. Addressing mental health and substance use before they escalate, coupled with holistic care strategies, can prevent homelessness and mitigate broader societal instability [8,45].

In summary, a focus on social sustainability for veterans requires integrated, adaptive policies that address the unique needs of this population. Tailored interventions, such as workforce reintegration, mental health support, and housing programs that incorporate cultural competency, will foster long-term stability and resilience. As the veteran population continues to diversify, policies must be flexible and data-driven, ensuring that interventions remain effective in addressing evolving demographic and clinical needs [38,39,45]. Strengthening these support systems ensures that veterans receive the care they need to maintain stable housing, employment, and social connections, fostering a more resilient and inclusive society.

## 5. Conclusions

This study offers valuable insights into the sociodemographic and clinical differences between homeless veterans and non-veterans, informing evidence-based service delivery. Veterans tend to be older, male, and have higher education and employment rates, but face unique challenges, including a higher prevalence of PTSD, depression, and co-occurring mental health conditions. In contrast, non-veterans struggle more with substance use, highlighting the need for targeted interventions. The findings emphasize expanding trauma-informed care, integrating mental health and substance use treatments, and implementing long-term housing programs for veterans, while prioritizing substance use disorder treatment for non-veterans.

Addressing homelessness through tailored interventions aligns with social sustainability principles, ensuring equitable access to mental health care, housing, and employment opportunities. Key implications include targeted support for vulnerable populations, inclusive economic participation, workforce reintegration [49], and long-term care approaches for chronic mental health and substance use disorders, namely the “Housing First” model, which emphasizes securing stable housing as the first step before tackling issues such as addiction or mental health concerns. Future research should explore the feasibility and implementation of a Housing First model tailored to the veteran community. Key areas of investigation include the practical design of such an approach, the policy frameworks necessary to support it, and the integration of tenancy sustainment strategies alongside access to critical health and support services. Addressing these questions will be essential to understanding how Housing First can effectively respond to the unique needs of veterans. Preventative measures are essential to reducing homelessness and its societal costs. Ultimately, fostering resilience, equity, and well-being requires not only addressing immediate needs but also creating sustainable, inclusive support systems, particularly for marginalized groups like homeless veterans.

## Figures and Tables

**Table 1:** Sample sociodemographic frequencies (*N* = 102,656).

Sociodemographic			
**Age**		N	%
4	18–20 years	2758	2.7
5	21–24 years	6114	6.0
6	25–29 years	12,660	12.3
7	30–34 years	13,801	13.4
8	35–39 years	13,598	13.2
9	40–44 years	11,478	11.2
10	45–49 years	11,700	11.4
11	50–54 years	11,857	11.6
12	55–59 years	10,210	9.9
13	60–64 years	5428	5.3
14	65 years and older	3052	3.0
−9	Missing/unknown/not collected/invalid	5	0.0
Gender		N	%
1	Male	62,111	60.5
2	Female	40,545	39.5
Race		N	%
1	American Indian/Alaska Native	3334	3.2
2	Asian	557	0.5
3	Black or African American	25,408	24.8
4	Native Hawaiian or Other Pacific Islander	298	0.3
5	White	62,275	60.7
6	Some other race alone/two or more races	7233	7.0
−9	Missing/unknown/not collected/invalid	3551	3.5
Education		N	%
1	Special education	396	0.4
2	0 to 8	7794	7.6
3	9 to 11	18,084	17.6
4	12 (or GED)	40,957	39.9
5	More than 12	18,076	17.6
−9	Missing/unknown/not collected/invalid	17,349	16.9
Marital status		N	%
1	Never married	57,144	55.7
2	Now married	6562	6.4
3	Separated	6915	6.7
4	Divorced, widowed	19,205	18.7
−9	Missing/unknown/not collected/invalid	12,830	12.5
Competitive employment status (aged 16 years and older)		N	%
1	Full-time	3586	3.5
2	Part-time	4070	4.0
3	Employed full-time/part-time not differentiated	589	0.6
4	Unemployed	47,200	46.0
5	Not in labor force	31,052	30.2
−9	Missing/unknown/not collected/invalid	16,159	15.7
Veteran status		N	%
1	Yes	6295	6.1
2	No	96,361	93.9

**Table 2:** Diagnosis differences between homeless veterans and homeless non-veterans (*N =* 102,656).

		Total (*N* = 102,656)		Veterans (*n* = 6295)		Non-Veterans (*n* = 96,361)				
SMI/SED Status		*n*	%	*n*	%	*n*	%	χ^2^	*p*	Ф
1	Serious mental illness (SMI)	76,400	74.4	5186	82.4	71,214	73.9	223.21	<0.001	0.05
2	Serious emotional disturbance (SED) and/or at risk for SED	17,256	16.8	728	11.6	16,528	17.2			
−9	Missing/unknown/not collected/invalid	8000	8.8	381	6.1	8619	8.90			
Mental Health Diagnosis One		*n*	%	n	%	n	%	χ^2^	*p*	Ф
1	Trauma- and stressor-related disorders	12,224	11.9	744	11.8	11,480	11.9	264.42	<0.001	0.05
2	Anxiety disorders	6608	6.4	303	4.8	6305	6.5			
3	Attention deficit/hyperactivity disorder (ADHD)	600	0.6	22	0.3	578	0.6			
4	Conduct disorders	151	0.1	5	0.1	146	0.2			
5	Delirium dementia	171	0.2	13	0.2	158	0.2			
6	Bipolar disorders	13,829	13.5	819	13.0	13,010	13.5			
7	Depressive disorders	27,299	26.6	1919	30.5	25,380	26.3			
8	Oppositional defiant disorders	74	0.1	2	0.0	72	0.1			
9	Pervasive developmental disorders	72	0.1	4	0.1	68	0.1			
10	Personality disorders	1159	1.1	47	0.7	1112	1.2			
11	Schizophrenia or other psychotic disorders	16,415	16.0	1219	19.4	15,196	15.8			
12	Alcohol or substance use disorders	10,516	10.2	366	5.8	10,150	10.5			
13	Other disorders/conditions	4957	4.8	285	4.5	4672	4.8			
−9	Missing/unknown/not collected/invalid/no or deferred diagnosis	8581	8.4	547	8.7	8034	8.3			
Mental Health Diagnosis Two		*n*	%	n	%	n	%	χ^2^	*p*	Ф
1	Trauma- and stressor-related disorders	5627	5.5	215	3.4	5412	5.6	632.40	<0.001	0.08
2	Anxiety disorders	7162	7.0	219	3.5	6943	7.2			
3	Attention deficit/hyperactivity disorder (ADHD)	891	0.9	26	0.4	865	0.9			
4	Conduct disorders	121	0.1	2	0.0	119	0.1			
5	Delirium dementia	101	0.1	11	0.2	90	0.1			
6	Bipolar disorders	2103	2.0	71	1.1	2032	2.1			
7	Depressive disorders	4956	4.8	190	3.0	4766	4.9			
8	Oppositional defiant disorders	46	0.0	3	0.0	46	0.0			
9	Pervasive developmental disorders	91	0.1	65	1.0	88	0.1			
10	Personality disorders	1937	1.9	54	0.9	1872	1.9			
11	Schizophrenia or other psychotic disorders	1638	1.6	224	3.6	1584	1.6			
12	Alcohol or substance use disorders	5827	5.7	147	2.3	5603	5.8			
13	Other disorders/conditions	4214	4.1	215	3.4	4067	4.2			
−9	Missing/unknown/not collected/invalid/no or deferred diagnosis	67,942	66.2	5068	80.5	62,874	65.2			
Mental Health Diagnosis Three		*n*	%	n	%	n	%	χ^2^	*p*	Ф
1	Trauma- and stressor-related disorders	1303	1.3	46	0.7	1257	1.3	158.52	<0.001	0.04
2	Anxiety disorders	1669	1.6	52	0.8	1617	1.7			
3	Attention deficit/hyperactivity disorder (ADHD)	471	0.5	13	0.2	458	0.5			
4	Conduct disorders	53	0.1	3	0.0	50	0.1			
5	Delirium dementia	27	0.0	2	0.0	25	0.0			
6	Bipolar disorders	569	0.6	14	0.2	555	0.6			
7	Depressive disorders	975	0.9	26	0.4	949	1.0			
8	Oppositional defiant disorders	16	0.0	0	0.0	16	0.0			
9	Pervasive developmental disorders	39	0.0	3	0.0	36	0.0			
10	Personality disorders	892	0.9	20	0.3	872	0.9			
11	Schizophrenia or other psychotic disorders	398	0.4	8	0.1	390	0.4			
12	Alcohol or substance use disorders	1667	1.6	83	1.3	1584	1.6			
13	Other disorders/conditions	897	0.9	22	0.3	875	0.9			
−9	Missing/unknown/not collected/invalid/no or deferred diagnosis	93,680	91.3	6003	95.4	87,677	91.0			
Number of mental health diagnoses reported		*n*	%	n	%	n	%	χ^2^	*p*	Ф
0	0	8581	8.4	547	8.7	8034	8.3	636.56	<0.001	0.08
1	1	59,361	57.8	4521	71.8	54,840	56.9			
2	2	25,738	25.1	935	14.9	24,803	25.7			
3	3	8976	8.7	292	4.6	8684	9.0			
Substance use diagnosis		*n*	%	n	%	n	%	χ^2^	*p*	Ф
1	Alcohol-induced disorder	293	0.3	20	0.3	273	0.3	373.73	<0.001	0.06
2	Alcohol intoxication	1108	1.1	54	0.9	1054	1.1			
3	Substance-induced disorder	2434	2.4	134	2.1	2300	2.4			
4	Alcohol dependence	5754	5.6	302	4.8	5452	5.7			
5	Cocaine dependence	1453	1.4	78	1.2	1375	1.4			
6	Cannabis dependence	1885	1.8	55	0.9	1830	1.9			
7	Opioid dependence	6353	6.2	176	2.8	6177	6.4			
8	Other substance dependence	4231	4.1	126	2.0	4105	4.3			
9	Alcohol abuse	1560	1.5	57	0.9	1503	1.6			
10	Cocaine abuse	591	0.6	32	0.5	559	0.6			
11	Cannabis abuse	1572	1.5	57	0.9	1515	1.6			
12	Opioid abuse	481	0.5	17	0.3	464	0.5			
13	Other substance related conditions	1711	1.7	93	1.5	1618	1.7			
−9	Missing/unknown/not collected/invalid/no or deferred diagnosis	73,230	71.3	5094	80.9	68,136	70.7			
Substance use problem		*n*	%	n	%	n	%	χ^2^	*p*	Ф
1	Yes	59,403	57.9	4710	74.8	54,693	56.8	809.02	<0.001	0.09
2	No	35,847	34.9	1394	22.1	34,453	35.8			
−9	Missing/unknown/not collected/invalid	7406	7.2	191	3.0	7215	7.5			

**Table 3:** Differences in other disorders between homeless veterans and homeless non-veterans (N = 102,656).

		Total		Veterans		Non-Veterans				
		(*N* = 102,656)		(*n* = 6295)		(*n* = 96,361)				
		N	%	N	%	N	%	χ^2^	*p*	Ф
Trauma- or stressor-related disorder reported	No	83,502	81.3	5290	84.0	78,212	81.2	30.05	<0.001	0.02
	Yes	19,154	18.7	1005	16.0	18,149	18.8			
Anxiety disorder reported	No	87,217	85.0	5721	90.9	81,496	84.6	184.01	<0.001	0.04
	Yes	15,439	15.0	574	9.1	14,865	15.4			
Attention deficit/hyperactivity disorder reported	No	100,694	98.1	6234	99.0	94,460	98.0	31.76	<0.001	0.02
	Yes	1962	1.9	61	1.0	1901	2.0			
Conduct disorder reported	No	102,331	99.7	6285	99.8	96,046	99.7	5.29	<0.001	0.01
	Yes	325	0.3	10	0.2	315	0.3			
Delirium/dementia disorder reported	No	102,357	99.7	6269	99.6	96,088	99.7	3.42	0.064	0.01
	Yes	299	0.3	26	0.4	273	0.3			
Bipolar disorder reported	No	86,155	83.9	5391	85.6	80,764	83.8	14.60	<0.001	0.01
	Yes	16,501	16.1	904	14.4	15,597	16.2			
Depressive disorder reported	No	69,426	67.6	4160	66.1	65,266	67.7	7.32	0.007	−0.01
	Yes	33,230	32.4	2135	33.9	31,095	32.3			
Oppositional defiant disorder reported	No	102,520	99.9	6293	100.0	96,227	99.9	5.14	0.023	0.01
	Yes	136	0.1	2	0.0	134	0.1			
Pervasive developmental disorder reported	No	102,454	99.8	6285	99.8	96,169	99.8	0.49	0.483	0.01
	Yes	202	0.2	10	0.2	192	0.2			
Personality disorder reported	No	98,668	96.1	6163	97.9	92,505	96.0	57.41	<0.001	0.02
	Yes	3988	3.9	132	2.1	3856	4.0			
Schizophrenia or other psychotic disorder reported	No	84,205	82.0	5014	79.7	79,191	82.2	25.68	<0.001	−0.02
	Yes	18,451	18.0	1281	20.3	17,170	17.8			
Alcohol or substance-related disorder reported	No	84,646	82.5	5622	89.3	79,024	82.0	217.72	<0.001	0.05
	Yes	18,010	17.5	673	10.7	17,337	18.0			
Other mental disorder reported	No	92,589	90.2	5841	92.8	86,748	90.0	51.04	<0.001	0.02
	Yes	10,067	9.8	454	7.2	9613	10.0			

**Table 4:** Binary logistic regression in predicting veteran status in American homeless.

	B	S.E.	Wald	Df	*p*	OR	95% CI for OR	
							Lower	Upper
Age	0.138	0.006	524.313	1	<0.001	1.147	1.134	1.161
Gender—Female	−0.877	0.033	721.866	1	<0.001	0.416	0.390	0.443
Race			71.304	6	<0.001			
Race—Black or African American	0.285	0.108	7.034	1	0.008	1.330	1.077	1.643
Race—White	0.415	0.105	15.529	1	<0.001	1.514	1.232	1.861
Race—Some other race alone/two or more races	0.267	0.116	5.303	1	0.021	1.306	1.041	1.639
Education			384.235	5	<0.001			
Education—12 years	0.585	0.056	107.043	1	<0.001	1.794	1.606	2.004
Education—more than 12 years	0.743	0.060	151.341	1	<0.001	2.102	1.868	2.366
Marital Status			50.104	4	<0.001			
Marital status—never married	0.277	0.068	16.760	1	<0.001	1.319	1.155	1.506
Marital status—now married	0.462	0.083	31.123	1	<0.001	1.588	1.350	1.868
Marital status—separated	0.371	0.083	19.899	1	<0.001	1.450	1.231	1.706
Marital status—divorced. widowed	0.426	0.071	35.711	1	<0.001	1.531	1.332	1.761
Competitive Employment Status			153.870	5	<0.001			
Competitive employment status—full time	0.611	0.079	59.978	1	<0.001	1.842	1.578	2.149
Competitive employment status—part time	0.220	0.083	7.075	1	0.008	1.246	1.060	1.465
Competitive employment status—unemployed	0.300	0.050	35.485	1	<0.001	1.350	1.223	1.491
SMI/SED Status			141.046	2	<0.001			
Serious mental illness (SMI)	0.640	0.064	98.382	1	<0.001	1.896	1.671	2.151
Serious emotional disturbance (SED) and/or at risk for SED	0.264	0.071	13.992	1	<0.001	1.302	1.134	1.495
Mental Health Diagnosis One			24.655	13	0.026			
Trauma- and stressor-related disorders	0.183	0.066	7.760	1	0.005	1.201	1.056	1.366
Schizophrenia or other psychotic disorders	0.203	0.062	10.835	1	<0.001	1.225	1.086	1.383
Mental Health Diagnosis Two			263.304	13	<0.001			
Trauma- and stressor-related disorders	−0.434	0.077	31.721	1	<0.001	0.648	0.557	0.753
Anxiety disorders	−0.785	0.076	107.804	1	<0.001	0.456	0.393	0.529
Attention deficit/hyperactivity disorder (ADHD)	−0.760	0.205	13.764	1	<0.001	0.468	0.313	0.699
Bipolar disorders	−0.615	0.127	23.372	1	<0.001	0.541	0.421	0.694
Depressive disorders	−0.481	0.082	34.110	1	<0.001	0.618	0.526	0.726
Personality disorders	−0.714	0.130	30.026	1	<0.001	0.489	0.379	0.632
Schizophrenia or other psychotic disorders	−0.700	0.144	23.744	1	<0.001	0.496	0.375	0.658
Alcohol or substance use disorders	−0.699	0.073	92.479	1	<0.001	0.497	0.431	0.573
Mental Health Diagnosis Three—Personality disorders	−0.566	0.232	5.977	1	0.014	0.568	0.361	0.894
Substance Use Diagnosis			548.973	13	<0.001			
Alcohol-induced disorder	−0.582	0.238	5.973	1	0.015	0.559	0.350	0.891
Alcohol intoxication	−0.339	0.150	5.145	1	0.023	0.712	0.531	0.955
Substance-induced disorder	−0.443	0.101	19.231	1	<0.001	0.642	0.527	0.783
Alcohol dependence	−0.804	0.065	154.438	1	<0.001	0.447	0.394	0.508
Cocaine dependence	−0.728	0.121	36.295	1	<0.001	0.483	0.381	0.612
Cannabis dependence	−1.113	0.140	63.044	1	<0.001	0.329	0.250	0.433
Opioid dependence	−1.143	0.082	192.661	1	<0.001	0.319	0.271	0.375
Other substance dependence	−1.140	0.095	145.199	1	<0.001	0.320	0.266	0.385
Alcohol abuse	−1.089	0.138	61.820	1	<0.001	0.337	0.257	0.442
Cocaine abuse	−0.666	0.187	12.732	1	<0.001	0.514	0.356	0.741
Cannabis abuse	−0.910	0.138	43.194	1	<0.001	0.403	0.307	0.528
Opioid abuse	−0.894	0.252	12.611	1	<0.001	0.409	0.250	0.670
Other substance related conditions	−0.445	0.112	15.730	1	<0.001	0.641	0.514	0.798
Constant	−6.361	0.163	1526.279	1	0.000	0.002		

Note. Inserted variables: Age, Gender, Race, Education, Marital Status, Competitive Employment Status, SMI/SED Status, Mental Health Diagnosis One, Two, and Three, Substance Use Diagnosis. Β = estimated value of the regression coefficient; SE = Standard error; Wald = Wald statistic; df = degrees of freedom; *p* = level of significance; OR = Odds Ratio; OR 95% CI = Odds ratio with a 95% confidence interval.

## Data Availability

The data used in this study were provided by the Center for Behavioral Health Statistics and Quality, Substance Abuse and Mental Health Services Administration (SAMHSA), USA, under the designation “Mental Health Client-Level Data (MH-CLD): 2019”. MH-CLD is a public database that provides demographic and mental health characteristics for clients who have used mental health services in facilities that report to individual state administrative data systems (https://www.samhsa.gov/data/data-we-collect/mh-cld-mental-health-client-level-data/datafiles) (accessed on 1 September 2024).

## References

[1] Omerov P., Craftman Å.G., Mattsson E., Klarare A. (2020). Homeless persons’ experiences of health- and social care: A systematic integrative review. Health Soc. Care Community.

[2] Gulati G., Keating N., O’Neill A., Delaunois I., Meagher D., Dunne C.P. (2019). The prevalence of major mental illness, substance misuse and homelessness in Irish prisoners: Systematic review and meta-analyses. Ir. J. Psychol. Med..

[3] Iwundu C.N., Chen T.A., Edereka-Great K., Businelle M.S., Kendzor D.E., Reitzel L.R. (2020). Mental Illness and Youth-Onset Homelessness: A Retrospective Study among Adults Experiencing Homelessness. Int. J. Environ. Res. Public Health.

[4] European Federation of National Organizations (FEANTSA), European Observatory on Homelessness (EOH) (2018). ETHOS: The European Typology of Homelessness and Housing Exclusion [Report]. FEANTSA. https://www.feantsa.org/en/toolkit/2005/04/01/ethos-typology-on-homelessness-and-housing-exclusion.

[5] (2009). The McKinney-Vento Homeless Assistance Act, as Amended by S. 896, The Homeless Emergency Assistance and Rapid Transition to Housing (HEARTH) Act of 2009, Pub. L. No. 111-22, 123 Stat. 1663. https://www.hudexchange.info/homelessness-assistance/hearth-act/.

[6] Schinka J., Byrne T. (2018). Aging and Life Expectancy in Homeless Veterans: Nine Questions. https://hdl.handle.net/2144/37729.

[7] Lowman C.A., Sheetz R.L., Ritchie E.C., Llorente M.D. (2021). VA Clinical Services: The Key to Achieving Stability and Sustainment for Homeless Veterans. Clinical Management of the Homeless Patient.

[8] Nichter B., Tsai J., Pietrzak R.H. (2023). Prevalence, correlates, and mental health burden associated with homelessness in US military veterans. Psychol. Med..

[9] Smith C., Castañeda E. (2020). Sick Enough? Mental Illness and Service Eligibility for Homeless Individuals at the Border. Soc. Sci..

[10] Amore K., Baker M., Howden-Chapman P. (2011). The ETHOS Definition and Classification of Homelessness: An Analysis. Eur. J. Homelessness.

[11] Substance Abuse and Mental Health Services Administration, Center for Behavioral Health Statistics and Quality (2021). Mental Health Client-Level Data 2019.

[12] Bretherton J., Pleace N. (2023). The Routledge Handbook of Homelessness.

[13] Henry M., Mahathey A., Morrill T., Robinson A., Shivji A., Watt R. (2018). The 2018 Annual Homeless Assessment Report (AHAR) to Congress: Part 1: Point-In-Time Estimates of Homeless. https://www.huduser.gov/portal/sites/default/files/pdf/2018-AHAR-Part-1.pdf.

[14] Kuhn R., Culhane D.P. (1998). Applying cluster analysis to test a typology of homelessness by pattern of shelter utilization: Results from the analysis of administrative data. Am. J. Community Psychol..

[15] Walters J.E., Lucero J., Wever C., Post A. (2021). Landlord Perceptions on Homelessness in Northern Utah. Soc. Sci..

[16] Culhane D. (1990). Chronic homelessness. J. Soc. Issues.

[17] Culhane D.P. (2008). The cost of homelessness: A perspective from the United States. Eur. J. Homelessness.

[18] Culhane D.P., Metraux S., Park J.M., Schretzman M., Valente J. (2007). Testing a typology of family homelessness based on patterns of public shelter utilization in four US jurisdictions: Implications for policy and program planning. Hous. Policy Debate.

[19] Markowitz F.E., Syverson J. (2019). Race, Gender, and Homelessness Stigma: Effects of Perceived Blameworthiness and Dangerousness. Deviant Behav..

[20] (2023). Veteran. https://www.merriam-webster.com/dictionary/veteran.

[21] Tsai J., Pietrzak R.H., Szymkowiak D. (2021). The problem of veteran homelessness: An update for the new decade. Am. J. Prev. Med..

[22] Tsai J., Mitchell L., Nakashima J., Blue-Howells J. (2023). Unmet needs of homeless U.S. veterans by gender and race/ethnicity: Data from five annual surveys. Psychol. Serv..

[23] Cypel Y., Schnurr P.P., Schneiderman A.I., Culpepper W.J., Akhtar F.Z., Morley S.W., Fried D.A., Ishii E.K., Davey V.J. (2022). The mental health of Vietnam theater veterans-the lasting effects of the war: 2016-2017 Vietnam Era Health Retrospective Observational Study. J. Trauma. Stress.

[24] Wood L., Flatau P., Seivwright A., Wood N. (2022). Out of the trenches; prevalence of Australian veterans among the homeless population and the implications for public health. Aust. N. Z. J. Public Health.

[25] Tsai J., Byrne T.H. (2019). National Utilization Patterns of Veterans Affairs Homelessness Programs in the Era of Housing First. Psychiatr. Serv..

[26] Tsai J., Link B., Rosenheck R.A., Pietrzak R.H. (2016). Homelessness among a nationally representative sample of US veterans: Prevalence, service utilization, and correlates. Soc. Psychiatry Psychiatr. Epidemiol..

[27] National Institute of Mental Health (2024). Mental Illness. https://www.nimh.nih.gov/health/statistics/mental-illness.

[28] Wiersma D. (2006). Needs of people with severe mental illness. Acta Psychiatr. Scand. Suppl..

[29] World Health Organization (2001). The World Health Report: 2001: Mental Health: New Understanding, New Hope. https://iris.who.int/handle/10665/42390.

[30] Ruggeri M., Leese M., Thornicroft G., Bisoffi G., Tansella M. (2000). Definition and prevalence of severe and persistent mental illness. Br. J. Psychiatry J. Ment. Sci..

[31] Weisbrot D.M., Carlson G.A. (2021). When No Diagnosis "Fits": Diagnostically Homeless and Emotionally Dysregulated Children and Adolescents. Child Adolesc. Psychiatr. Clin. N. Am..

[32] Zhao E. (2022). The key factors contributing to the persistence of homelessness. Int. J. Sustain. Dev. World Ecol..

[33] Ding K., Slate M., Yang J. (2018). History of co-occurring disorders and current mental health status among homeless veterans. BMC Public Health.

[34] Smartt C., Prince M., Frissa S., Eaton J., Fekadu A., Hanlon C. (2019). Homelessness and severe mental illness in low- and middle-income countries: Scoping review. BJPsych Open.

[35] Metraux S., Cusack M., Byrne T.H., Hunt-Johnson N., True G. (2017). Pathways into homelessness among post-9/11-era veterans. Psychol. Serv..

[36] Crone B., Metraux S., Sbrocco T. (2022). Health service access among homeless veterans: Health access challenges faced by homeless African American veterans. J. Racial Ethn. Health Disparities.

[37] Tsai J., Kelton K. (2023). Service use and barriers to care among homeless veterans: Results from the national veteran homeless and other poverty experiences (nv-hope) study. J. Community Psychol..

[38] Bennett A., Crosse K., Ku M., Edgar N.E., Hodgson A., Hatcher S. (2022). Interventions to treat post-traumatic stress disorder (PTSD) in vulnerably housed populations and trauma-informed care: A scoping review. BMJ Open.

[39] Peter S.C., Halverson T.F., Blakey S.M., Pugh M.J., Beckham J.C., Calhoun P.S., Kimbrel N.A. (2023). The Veterans Health Administration’s integrated model of care increases accessibility and delivery of mental health services. Psychol. Serv..

[40] Gabrielian S., Koosis E.R., Cohenmehr J., Hellemann G., Tuepker A., Green M.F., Vazzano J.K., Young A.S. (2022). Factors associated with recovery from homelessness among veterans in permanent supportive housing. J. Community Psychol..

[41] Loubière S., Lemoine C., Boucekine M., Boyer L., Girard V., Tinland A., Auquier P., for the French Housing First Study Group (2022). Housing First for homeless people with severe mental illness: Extended 4-year follow-up and analysis of recovery and housing stability from the randomized Un Chez Soi d’Abord trial. Epidemiol. Psychiatr. Sci..

[42] Tsai J., Mehta K., Mongtomery A.E., Elbogen E., Hooshyar D. (2021). Changing demography of homeless adult populations. Perspect. Public Health.

[43] Israel B.S., Belcher A.M., Ford J.D. (2024). A harm reduction framework for integrated treatment of co-occurring opioid use disorder and trauma-related disorders. J. Dual Diagn..

[44] Stablein G.W., Hill B.S., Keshavarz S., Llorente M.D. (2021). Homelessness and substance use disorders. Clinical Management of the Homeless Patient: Social, Psychiatric, and Medical Issues.

[45] Cox C.B. (2015). Social Policy for an Aging Society: A Human Rights Perspective.

[46] Otiniano Verissimo A.D., Henley N., Gee G.C., Davis C., Grella C. (2023). Homelessness and discrimination among US adults: The role of intersectionality. J. Soc. Distress Homelessness.

[47] Olivet J., Wilkey C., Richard M., Dones M., Tripp J., Beit-Arie M., Yampolskaya S., Cannon R. (2021). Racial inequity and homelessness: Findings from the SPARC study. Ann. Am. Acad. Political Soc. Sci..

[48] Beydoun H.A., Mayno Vieytes C., Beydoun M.A., Lampros A., Tsai J. (2024). Correlates of six-month housing instability among US adults by veteran status: Exploratory study using data from the All of Us Program. PLoS ONE.

[49] Miklewska A. (2024). Supported employment–the way to integration and independence for people in a homelessness crisis. Humanit. Soc. Sci..

[50] Sprong M.E., Hollender H., Paul E., Gilbert J., Weber K., Garakani A., Buono F.D. (2023). Impact of substance use disorders on employment for veterans. Psychol. Serv..

[51] Spector A.L., Quinn K.G., McAuliffe T.L., DiFranceisco W., Bendixen A., Dickson-Gomez J. (2020). Health-related quality of life and related factors among chronically homeless adults living in different permanent supportive housing models: A cross-sectional study. Qual. Life Res. Int. J. Qual. Life Asp. Treat. Care Rehabil..

[52] Fang M.L., Canham S.L., Battersby L. (2023). Supporting intersecting cultural needs of gender and age by increasing cultural safety and humility for Housing First initiatives. BMC Public Health.

[53] Nielssen O.B., Stone W., Jones N.M., Challis S., Nielssen A., Elliott G., Burns N., Rogoz A., Cooper L.E., Large M.M. (2018). Characteristics of people attending psychiatric clinics in inner Sydney homeless hostels. Med. J. Aust..

[54] Moschion J., Johnson G. (2019). Homelessness and incarceration: A reciprocal relationship?. J. Quant. Criminol..

[55] Tweed E.J., Thomson R.M., Lewer D., Sumpter C., Kirolos A., Southworth P.M., Purba A.K., Aldridge R.W., Hayward A., Story A. (2021). Health of people experiencing co-occurring homelessness, imprisonment, substance use, sex work and/or severe mental illness in high-income countries: A systematic review and meta-analysis. J. Epidemiol. Community Health.

[56] Neisler J., Shree S., Reitzel L.R., Chen T.A., Kendzor D.E., Obasi E.M., Wrighting Q., Businelle M.S. (2019). Characterizing Alcohol Use Behaviors among Homeless Men and Women. Am. J. Health Behav..

